# Imbalance of Circulating Innate Lymphoid Cell Subpopulations in Patients With Septic Shock

**DOI:** 10.3389/fimmu.2019.02179

**Published:** 2019-09-20

**Authors:** Julien Carvelli, Christelle Piperoglou, Jeremy Bourenne, Catherine Farnarier, Nathalie Banzet, Clemence Demerlé, Marc Gainnier, Frédéric Vély

**Affiliations:** ^1^APHM, Service de Médecine Intensive et Réanimation, Réanimation Des Urgences, Hôpital la Timone, Marseille, France; ^2^CEReSS - Center for Studies and Research on Health Services and Quality of Life EA3279, Aix-Marseille University, Marseille, France; ^3^APHM, Hôpital de la Timone, Service d'Immunologie, Marseille Immunopôle, Marseille, France; ^4^Aix Marseille Univ, CNRS, INSERM, CIML, Marseille, France

**Keywords:** sepsis, septic shock, immunosuppression, sepsis-induced immunosuppression, innate lymphoid cells (ILC), NK cells, lymphopenia

## Abstract

**Background:** Septic shock, a major cause of death in critical care, is the clinical translation of a cytokine storm in response to infection. It can be complicated by sepsis-induced immunosuppression, exemplified by blood lymphopenia, an excess of circulating Treg lymphocytes, and decreased HLA-DR expression on circulating monocytes. Such immunosuppression is associated with secondary infections, and higher mortality. The effect of these biological modifications on circulating innate lymphoid cells (ILCs) has been little studied.

**Methods:** We prospectively enrolled patients with septic shock (Sepsis-3 definition) in the intensive care unit (ICU) of Timone CHU Hospital. ICU controls (trauma, cardiac arrest, neurological dysfunction) were recruited at the same time (NCT03297203). We performed immunophenotyping of adaptive lymphocytes (CD3^+^ T cells, CD19^+^ B cells, CD4^+^CD25^+^FoxP3^+^ Treg lymphocytes), ILCs (CD3^−^CD56^+^ NK cells and helper ILCs – ILC1, ILC2, and ILC3), and monocytes by flow cytometry on fresh blood samples collected between 24 and 72 h after admission.

**Results:** We investigated adaptive and innate circulating lymphoid cells in the peripheral blood of 18 patients in septic shock, 15 ICU controls, and 30 healthy subjects. As expected, the peripheral blood lymphocytes of all ICU patients showed lymphopenia, which was not specific to sepsis, whereas those of the healthy volunteers did not. Circulating CD3^+^ T cells and CD3^−^CD56^+^ NK cells were mainly concerned. There was a tendency toward fewer Treg lymphocytes and lower HLA-DR expression on monocytes in ICU patients with sepsis. Although the ILC1 count was higher in septic patients than healthy subjects, ILC2, and ILC3 counts were lower in both ICU groups. However, ILC3s within the total ILCs were overrepresented in patients with septic shock. The depression of immune responses has been correlated with the occurrence of secondary infections. We did not find any differences in ILC distribution according to this criterion.

**Conclusion:** All ICU patients exhibit lymphopenia, regardless of the nature (septic or sterile) of the initial medical condition. Specific distribution of circulating ILCs, with an excess of ILC1, and a lack of ILC3, may characterize septic shock during the first 3 days of the disease.

## Introduction

Sepsis and septic shock are major public health concerns. In the absence of therapeutic advances, septic shock is a leading cause of mortality in critically ill patients (1). Septic shock corresponds to an intense and uncontrolled systemic immune response to a bacterial or fungal infection (2). In the first hours, the activation of innate immunity leads to cytokine storm syndrome (CSS), which can lead to multiple organ failure and early death. In some survivors, secondary immune dysfunction can occur. Immunosuppression can lead to secondary infections, such as ventilator-associated pneumonia (VAP), which increases morbidity, and mortality (3). Immunosuppression is not specific to sepsis or septic shock in critical care. It involves both innate and adaptive immunity. The phagocytosis of peripheral blood neutrophils can be altered, leading to VAP (4), as reduced IFN-γ production by natural killer (NK) cells is associated with CMV reactivation (5). Activation markers, such as HLA-DR (MHC class II), on circulating monocytes are underexpressed in septic patients (6), especially in the later stages of the disease (> 72 h) (7), and this is associated with secondary infections, and mortality. Lymphopenia is also common in patients admitted for septic shock, with the loss of splenic CD4^+^ T cells (autopsy findings), and reduced levels of circulating B lymphocytes (8). Conversely, circulating regulatory T cells (Tregs) are always overrepresented 3 to 7 days after diagnosis of the infection (9), and promote anti-inflammatory responses during the second phase of sepsis (10).

Innate lymphoid cells (ILCs) are innate lymphocytes. ILC subgroups are defined according to their expression of key transcription factors and cytokine production, based on their similarity to T cells, and T helper (T_H_) cell subsets. Thus, ILCs can be classified as “cytotoxic” ILCs (bona fide NK cells) or as “helper” ILCs (ILC1, ILC2, and ILC3) (11, 12). Helper ILCs represent 0.1% of all circulating lymphocytes (13). The ILC1, ILC2, and ILC3 subsets are present mainly as sedentary cells in tissues, in which they can be maintained by self-renewal (14). Nevertheless, ILC subsets are detectable in human peripheral blood by flow cytometry by excluding lineage (Lin) positive cells (including CD3^+^ T cells, CD19^+^ B cells, CD94^+^ NK cells, and CD14^+^ monocyte/myeloid cells), and gating on cells that express the IL-7 receptor (CD127; **Figure 3**). Helper ILC1s require T-bet for development and produce IFN-γ as their main effector cytokine. ILC2s depend on GATA-3 and produce “Th2” cytokines (IL-4, IL-5, IL-9, and IL-13). They express CRTH2, a marker used in the gating strategy. ILC3s depend on RORγt, secrete “Th17” cytokines, such as IL-17, or “Th22” cytokines, such as IL-22, and express CD117 (gating strategy). Many studies have suggested an important role for helper ILCs in immunity, particularly in mouse models. A review on the role of ILCs in inflammatory diseases has been recently published (15). ILC1s are pro-inflammatory cells and may be involved in the pathogenesis of chronic obstructive pulmonary disease (16) and Crohn's disease (17). ILC2s may be involved in atopic diseases (18) and may promote fibrosis (19). ILC3s may be critical effector cells in psoriasis (20). ILC biology has never been well-studied in sepsis, even if they appear to be redundant for protective immunity in humans when T-cell, and B-cell functions are preserved (13). Here, we prospectively evaluated circulating immune cells by flow cytometry, especially circulating ILCs, and their three subsets during the early phase of septic shock, defined as the first 3 days after diagnosis of the infection.

## Materials and Methods

### Patients

Patients with septic shock in the Intensive Care Unit (ICU) of Timone CHU Hospital (Réanimation des Urgences, AP-HM Marseille, France) were prospectively enrolled between June and December 2017. According to the Sepsis-3 definition (21), septic shock was considered to be a bacterial infection responsible for arterial hypotension or hyperlactatemia without hypovolemia. All patients required norepinephrine infusion after adequate fluid resuscitation. Hydrocortisone was systemically added as symptomatic therapeutic support for these patients (200 mg/day) (22, 23). First, we compared septic patients to other patients hospitalized in the ICU for cardiac arrest, trauma, or neurological dysfunction (stroke, status epilepticus). These patients had no sign of infection on the day of inclusion. Exclusion criteria were age < 18 years, life expectancy < 48 h, and bone-marrow aplasia (no circulating lymphocytes). Thirty healthy volunteers were recruited in our laboratory to determine normal values of circulating ILCs. All patient data are shown in Table 1. We compared all ICU patients who could have severe tissue injuries vs. all ICU patients with less severe injuries to assess the impact of “aggression intensity” on the depression of immune biomarkers. We used the SOFA (*Sepsis-related Organ Failure Assessment*) score, which adds organ dysfunctions (hemodynamic respiratory, hepatic, renal, neurological, and hematopoietic dysfunctions), to define severe tissue injuries. We arbitrarily defined a SOFA score ≥ 8 as corresponding to severe tissue damage. Patient characteristics according to the SOFA score are shown in Table 2. Finally, we compared critically ill patients who had a secondary infection during their stay in the ICU vs. patients who did not (patient data in Table 3) to assess the role of “biomarkers depression” in the occurrence of secondary infections. The study protocol was approved by the Committee for the Protection of Persons North-West II—France and the trial was registered online before initiation (NCT03297203). Written informed consent was obtained from each patient.

**Table 1:** Patient characteristics, ICU-septic patients vs. ICU controls.

	**ICU sepsis group *n* = 18**	**ICU control group *n* = 15**	**Healthy volunteers *n* = 30**	***p***
**Age—years**	57 [29-77]	50 [21–80]	32 [23–66]	0.12
**Gender—F/M**	7/11	5/10	19/11	0.74
**SOFA**	8 [4–15]	6 [2–17]	–	0.10
**SAPS II**	64 [28–76]	40 [25–85]	–	0.22
**Norepinephrine—mg/h**	3 [0.3–12]	1 [0–9]	–	**0.01**
**RRT—*****n*** **(%)**	1 (6%)	2 (13%)	–	0.44
**Mechanical ventilation—*****n*** **(%)**	12 (67%)	13 (87%)	–	0.18
**Mechanical ventilation (days)**	3 [0–40]	5 [0–120]	–	0.13
**Origin of sepsis**				
*Pneumonia*	6 (33%)	–	–	
*Abdominal infections*	5 (28%)	–	–	
*Urinary tract infections (UTI)*	4 (22%)	–	–	
*Catheter-related infections (CRI)*	2 (11%)	–	–	
*Soft tissue infection*	1 (6%)	–	–	
**Isolated bacteria at time of sepsis diagnosis—*****n*** **(%)**	14 (78%)	–	–	–
*S. pneumoniae*	1			
*S. oralis*	1			
*S. pyogenes*	1			
*H. influenza*	1			
*P. aeruginosae*	1			
*E. coli*	4			
*K. pneumonia*	3			
*K. oxytoca*	1			
*E. cloacae*	1			
**Secondary infections—*****n*** **(%)**	7 (39%)	6 (40%)	–	0.95
–Origin of infection				
*Ventilator-associated pneumonia (VAP)*	5^*^	5		
*C. difficile colitis*	1	0		
*Cholecystitis*	1	0		
*CRI*	0	1		
*CMV reactivation*	1^*^	0		
–Delay of occurrence since admission—days	7 [5–30]	6 [2–18]		0.38
Mortality day 60—*n* (%)	5 (28%)	6 (40%)	–	0.46
ICU length of stay (days)	6 [2–38]	7 [2–120]	–	0.72

*Variables are shown as medians [min-max]*.

*p values result from Mann-Withney U-test (continuous variables) or Chi squared test (categorical variables) and compare the **SOFA scores** (Sequential Organ Failure Assessment score—ranges from 0 to 24 points) of the 2 ICU groups: based on 6 different variables, one each for the respiratory (PaO2/FiO2 ratio and use of mechanical ventilation), cardiovascular (mean arterial pressure and use of vasopressors), hepatic (bilirubin), coagulation (platelets), renal (creatinine and urine output), and neurological (Glasgow coma scale) systems. Significant p values are in bold*.

***SAPS II** (Simplified Acute Physiology Score II—ranges from 0 to 163 points): based on age, chronic diseases, type of admission, heart rate, systolic blood pressure, temperature, Glasgow coma scale, PaO2/FiO2 ratio and use of mechanical ventilation, urine output, and biological parameters (urea, sodium, potassium, bicarbonate, bilirubin, and white blood cells). RRT: Renal-Replacement Therapy*.

* *Patient with CMV reactivation also had a bacterial VAP*.

**Table 2:** Patient characteristics, SOFA < 8 vs. SOFA ≥ 8.

	**SOFA <8 *n* = 14**	**SOFA ≥ 8 *n* = 19**	***p***
**Age—years**	56 [21–80]	56 [23–77]	0.91
**Gender—F/M**	4/10	8/11	0.42
**SOFA**	4 [2–7]	11 [8–17]	** <0.0001**
**SAPS II**	35 [26–70]	66 [25–85]	**0.0004**
**Type of admission**			
*Sepsis*	5	13	**0.03**
*Cardiac arrest*	2	4	0.6
*Trauma*	4	1	**0.04**
*Stroke*	1	1	0.8
*Status epilepticus*	1	0	0.2
*Voluntary overdose*	1	0	0.2
**Norepinephrine—mg/h**	0.2 [0–4]	3 [0.5–12]	** <0.0001**
**RRT—*****n*** **(%)**	0	3 (16%)	0.12
**Mechanical ventilation—*****n*** **(%)**	9 (64%)	16 (84%)	0.19
**Mechanical ventilation—days**	2 [0–120]	5 [0–40]	0.19
**Secondary infections—*****n*** **(%)**	6 (42%)	7 (37%)	0.73
–Origin of infection			
*Ventilator-associated pneumonia (VAP)*	5	5^*^	
*C. difficile colitis*	1	0	
*Cholecystitis*	0	1	
*CRI*	0	1	
*CMV recurrence*	0	1^*^	
–Time of occurrence since admission—days	7 [2–30]	6 [5–13]	0.91
**Mortality day 60—*****n*** **(%)**	2 (14%)	9 (47%)	**0.046**
**ICU length of stay—days**	4 [2–120]	7 [3–38]	0.14

*Variables are shown as medians [min-max]*.

*p values result from Mann-Withney U-test (continuous variables) or Chi squared test (categorical variables). Significant p values are in bold*.

***SOFA score** (Sequential Organ Failure Assessment score—ranges from 0 to 24 points)*.

***SAPS II** (Simplified Acute Physiology Score II—ranges from 0 to 163 points)*.

* *Patient with CMV recurrence had also a bacterial VAP. RRT: Renal-Replacement Therapy*.

**Table 3:** Patient characteristics, secondarily infected vs. others.

	**Secondarily infected patients *n* = 13**	**Others *n* = 20**	***p***
**Age—years**	57 [23–80]	56 [21–73]	0.26
**Gender—F/M**	5/8	7/13	0.84
**SOFA**	8 [4–17]	8 [2–16]	0.59
**SAPS II**	65 [28–77]	48 [25–85]	0.51
**Type of admission**			
*Sepsis*	7	11	0.95
*Cardiac arrest*	2	4	0.74
*Trauma*	3	2	0.30
*Stroke*	0	2	0.24
*Status epilepticus*	1	0	0.22
*Voluntary overdose*	0	1	0.41
**Norepinephrine—mg/h**	1.5 [0–12]	1.5 [0–8]	0.89
**RRT—*****n*** **(%)**	2 (15%)	1 (5%)	0.31
**Mechanical Ventilation—*****n*** **(%)**	11 (85%)	13 (65%)	0.22
**Mechanical Ventilation—days**	14 [0–40]	2 [0–120]	**0.004**
**Mortality day 60—*****n*** **(%)**	5 (38%)	6 (30%)	0.61
**ICU length of stay—days**	18 [2–60]	4 [2–120]	**0.001**

*Variables are shown as medians [min-max]*.

*p-values from the Mann-Whitney U-test (continuous variables) or Chi squared test (categorical variables). Significant p values are in bold*.

***SOFA score** (Sequential Organ Failure Assessment score—ranges from 0 to 24 points)*.

***SAPS II** (Simplified Acute Physiology Score II—ranges from 0 to 163 points). RRT: Renal-Replacement Therapy*.

### Immunophenotyping by Flow Cytometry

Biological analyses were performed on fresh blood during the 6 h following blood-sample collection. Blood samples were all collected between 24 and 72 h after ICU admission for all ICU patients. For patients with septic shock, ICU admission corresponded to the diagnosis of infection and the initiation of antibiotics. The first 3 days were considered to be the early phase of the disease based on the clinical and biological systemic inflammatory response (cytokine storm, hemodynamic impairment, multiple organ failure) (24, 25). Lymphocyte populations (total lymphocytes, CD3^+^ T cells, CD4^+^ T cells, CD8^+^ T cells, CD19^+^ B cells, and CD3^−^CD56^+^ NK cells) were quantified using 6-Color BD Multitest and BD Trucount Technologies (Becton Dickinson, Le Pont de Claix, France), according to the manufacturer's instructions. We also determined the HLA-DR expression on circulating monocytes. Whole blood was incubated with BD Quantibrite PE Anti-HLA-DR (clone L243)/Anti-Monocyte Stain (clone MΦP9; Becton Dickinson, San Jose, CA, USA). The analysis was performed on a BD FACSCanto II™ cytometer using BD FACS DIVA Software. The amount of antibody bound per cell (Ab/cell) was calculated by standardizing the HLA-DR geometric mean fluorescence intensity (MFI) of monocytes to BD Quantibrite phycoerythrin (PE) beads (BD CellQuest™ Software). Peripheral blood mononuclear cells (PBMCs) were immediately collected by MSL (Eurobio) density centrifugation. PBMCs were used to identify regulatory T cells, considered to be CD4^+^CD25^+^FoxP3^+^ lymphocytes. The following antibodies were used: AmCyan anti-CD3 (Clone SK7), APC-H7 anti-CD4 (clone SK3), PerCP-Cy5.5 anti-CD8 (clone SK1), APC anti-CD25 (clone 2A3), Alexa Fluor 488 anti-FoxP3 (clone 259D/C7), and Alexa Fluor 488 anti-IgG1 (isotype-matched controls) (BD Biosciences). PBMCs were also used to characterize ILCs in peripheral blood. We used a panel of conventional lineage markers (Lin: CD3, CD19, CD14, TCRαβ, TCRγδ, CD94, CD16, FcεRI, CD34, CD123, and CD303) and cell-surface expression of CD127, CD117, and CRTH2 to identify the ILC1 subset as Lin^−^ CD127^+^ CD117^−^ CRTH2^−^ cells, the ILC2 subset as Lin^−^ CD127^+^ CRTH2^+^ cells, and the ILC3 subset as Lin^−^ CD127^+^ CD117^+^ CRTH2^−^ cells, among the circulating lymphocytes. The following antibodies were used: FITC anti-CD3 (clone UCTH1), FITC anti-CD19 (clone HIB19), FITC anti-CD14 (clone M5E2), FITC anti-TCRαβ (clone T10B9), FITC anti-TCRγδ (clone B1), FITC anti-CD94 (clone HP-3D9), FITC anti-CD16 (clone 3G8), FITC anti-CD34 (clone 581/CD34), FITC anti-CD123 (clone 7G3) (BD Pharmigen), FITC anti-CD303 (clone AC144) (Miltenyi Biotec), FITC anti-FcεRI (AER-37) (Ebioscience), PE-C7 anti-CD127 (HIL-7R-M21) (BD Pharmigen), Alexa Fluor 647 anti-CD294/CRTH2 (clone BM16) (BD Horizon), and PE-Cy5 anti-CD117 (clone YB5.B8) (BD Pharmigen). Data was acquired using a BD LSRFortessa™ cytometer and data analysis was performed using FlowJo 10.2 Software.

### Statistical Analysis

The results are presented (text, tables, and figures) as the median [min, max]. For continuous variables, multiple group comparisons were analyzed using the Mann-Withney *U*-test for two groups and the Kruskal-Wallis test for more than two groups. For categorical variables, the Chi squared test was used. Statistical analyses were performed with Prism 6 (GraphPad Software, San Diego, CA, USA). Results were considered significant for a *p* < 0.05.

## Results

### Patient Characteristics (Tables 1−3)

Eighteen patients with septic shock were included (*ICU Sepsis Group*). The sources of infection and bacterial strains are detailed in Table 1. The ICU control group contained 15 patients (*ICU Control Group*): six cardiac arrests, five severe trauma, two strokes, one status epilepticus, and one voluntary overdose with benzodiazepine.

Patients with sepsis were slightly, but not significantly, older (57 years [29–77]) than the controls (50 years [21–80]; *p* = 0.12). Most patients were male (21/33), with no difference between the two groups (*p* = 0.74). The median SOFA score was higher for the *Sepsis Group* (8 [4–15]) than the ICU controls (6 [2–17]; *p* = 0.10). The same was true for the SAPSII (64 [28–76] vs. 40 [25–76], *p* = 0.22). According to the protocol for septic shock, all patients with sepsis received norepinephrine infusion with a median dosage of 3 mg/h [0.3–12], higher than for the controls, for whom norepinephrine infusion was from 1 mg/h [0–9] (*p* = 0.01). Only one patient in the *ICU Sepsis Group* required renal replacement therapy (RRT) vs. two in the control group (*p* = 0.44). Three quarters of the patients were mechanically ventilated (more in the sepsis group, 12 vs. 13, *p* = 0.18), with a median duration of mechanical ventilation of 3 days [0–40] in the sepsis group and 5 days [0–120] in the ICU control group (*p* = 0.13). Secondary infections in the ICU concerned seven patients with anterior septic shock and six ICU controls (*p* = 0.95). Secondary infections corresponded to 10 cases of VAP, one CRI, one case of cholecystitis, and one of *C. difficile* colitis. One patient with VAP also had *CMV* reactivation. These secondary infections occurred between day 2 and day 30 (median of seven days). Overall mortality at day 180 was 11/33 (33%) and was higher in the controls (6/15 vs. 5/18 deaths in the sepsis group, *p* = 0.46). The median length of stay in the ICU was 7 days and was nearly the same for the two groups (6 [2–38] for the sepsis group vs. 7 [2–20] for the control group, *p* = 0.72).

### Immunological Analysis (Table 4)

#### Conventional Blood Lymphocyte Immunophenotyping

**Figure 1:**
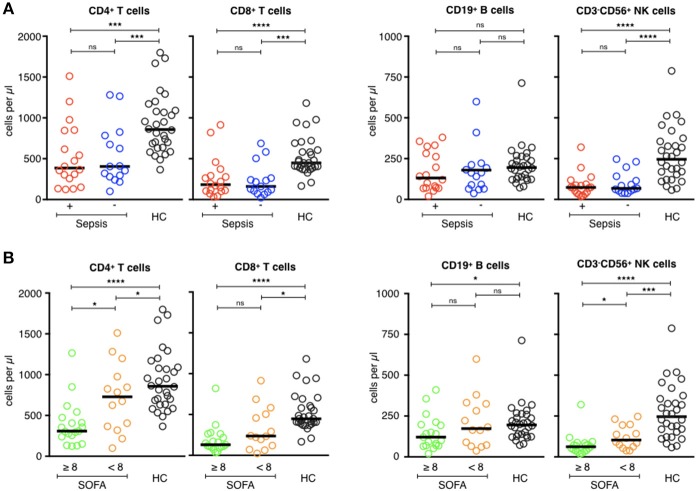
Phenotypic analysis of circulating lymphocyte subsets in ICU patients. **(A)** Comparison of helper T-cell, cytotoxic T-cell, B-cell, and NK-cell counts (cells/μl) of septic patients (red circles) with those of healthy controls (dark circles), and ICU patients without sepsis (blue circles). **(B)** Comparison of helper T-cell, cytotoxic T-cell, B-cell, and NK cell counts (cells/μl) of patients with severe tissue injuries (green circles) with those of healthy controls (dark circles), and patients with less severe lesions (orange circles). The bars show the median. Statistical analyses were performed using the Mann Whitney *U*-test. Differences were considered significant when *P* < 0.05: ^*^P < 0.05, ^***^P < 0.001, ^****^P < 0.0001. ns: not significant.

Most patients with septic shock develop lymphopenia (8, 26). Patients of the ICU groups developed lymphopenia, with no difference between the two groups (*p* = 0.90), whereas the lymphocyte counts of the healthy volunteers remained normal. The median lymphocyte count was 2,042/mm^3^ [708–3,606] for the healthy subjects vs. 992/mm^3^ [298–2,487] for the septic patients and 856/mm^3^ [298–2,246] for the ICU controls (*p* < 0.0001). Lymphopenia concerned all lymphocyte subsets, above all CD3^+^ T cells (TCD4^+^ and TCD8^+^), and CD3^−^CD56^+^ NK cells (Figure 1A). There was no statistical difference for the CD19^+^ B cell counts between the three groups. Patients with a secondary infection tended to have fewer circulating lymphocytes than those without, with no statistical difference (Table 4). The more extensive the tissue damage was (SOFA ≥ 8), the more pronounced was the deficit of circulating lymphocytes, with a correlation between a higher SOFA score, and lower lymphocyte counts (Rho (Spearman): −0.446 [IC 95%: −0.684, −0.121], *p* < 0.001). These results were only significant concerning CD4^+^ T cells and CD3^−^CD56^+^ NK cells (Figure 1B).

**Table 4:** Immunological analysis.

	**ICU sepsis *n* = 18**	**ICU controls *n* = 15**	**Healthy controls *n* = 30**	**Secondary infections *n* = 13**	**No secondary infection *n* = 20**	***p***	**SOFA <8 *n* = 14**	**SOFA ≥ 8 *n* = 19**	***p***
**Lymphocytes (/mm**^**3**^**)**	992 [298–2,487]	856 [298–2,246]	2,042 [708–3,606]	710 [319–1,517]	1,056 [298–2,487]	0.14	1,495 [298–2,487]	658 [298–1,987]	0.02
CD4^+^ T Cells	386 [124–1,510]	405 [100–1,265]	858 [366–1,731]	320 [124–845]	545 [100–1,510]	0.13	729 [100–1,510]	309 [124–1,265]	0.02
CD8^+^ T Cells	183 [32–914]	160 [23–687]	448 [166–1,181]	195 [23–687]	165 [32–914]	0.64	236 [23–914]	129 [32–820]	0.12
CD19^+^ B Cells	131 [20–380]	163 [37–599]	196 [72–713	150 [37–325]	141 [20–599]	0.54	173 [37–599]	121 [20–410]	0.31
CD3^−^CD56^+^ NK Cells	73 [18–320]	68 [38–247]	245 [57–787]	71 [18–198]	75 [31–320]	0.28	103 [38–247]	62 [18–320]	0.03
**Monocytes HLA-DR expression**									
MFI	2649 [959–8,752]	4882 [1,314–16,298]		3,702 [959–12,941]	3533 [1,048–16,298]	0.52	6176 [1,314–16,298]	3,479 [959–11,183]	0.08
Ab/Cell	5286 [2,089–16,832]	7,882 [2,760–24,756]		6,044 [2,089–24,756]	6,959 [2,257–23,846]	0.55	11,958 [2,760–24,756]	6856 [2,089–15,804]	0.05
**Regulatory T Cells** (CD4^+^CD25^+^FoxP3^+^)									
Absolute count (/mm^3^ of whole blood)	9 [1–80]	17 [5–64]		11 [3–38]	14 [1–80]	0.67	17 [1–80]	11 [3–34]	0.04
**ILC**									
**Total ILC** (/mL of whole blood)	1,293 [483–4,468]	1,242 [326–4,085]	1,632 [505–5,846]	1,120 [326–4,085]	1,277 [440–4,468]	0.7	1,031 [326–4,468]	1,264 [483–3,638]	0.98
**ILC1**									
Absolute count (/mL)	931 [282–3,478]	512 [133–2,978]	468 [178–1,980]	514 [209–2,978]	931 [133–3,478]	0.67	505 [133–3,127]	883 [282–3,478]	0.57
Percentage across ILCs	66 [32–96]	64 [27–83]	30 [13–71]	61 [27–77]	69 [29–96]	0.25	66 [27–82]	68 [32–96]	0.28
**ILC2**									
Absolute count (/mL)	239 [29–1,592]	147 [39–791]	584 [117–1,597]	294 [45–791]	161 [29–1,592]	0.3	211 [29–1,592]	147 [29–518]	0.60
Percentage across ILCs	20 [3–58]	14 [3–43]	28 [14–66]	21 [7–39]	13 [3–58]	0.14	20 [6–44]	14 [3–58]	0.20
**ILC3**									
Absolute count (/mL)	162 [41–469]	258 [71–1,460]	513 [83–2,424]	261 [41–800]	188 [41–1,460]	0.74	180 [59–1,460]	182 [41–644]	0.41
Percentage across ILCs	13 [2–31]	25 [8–49]	32 [12–55]	17 [5–49]	14 [2–48]	0.64	16 [3–49]	14 [3–43]	0.44

*Variables are shown as median [min-max]*.

#### Circulating Treg Lymphocytes

**Figure 2:**
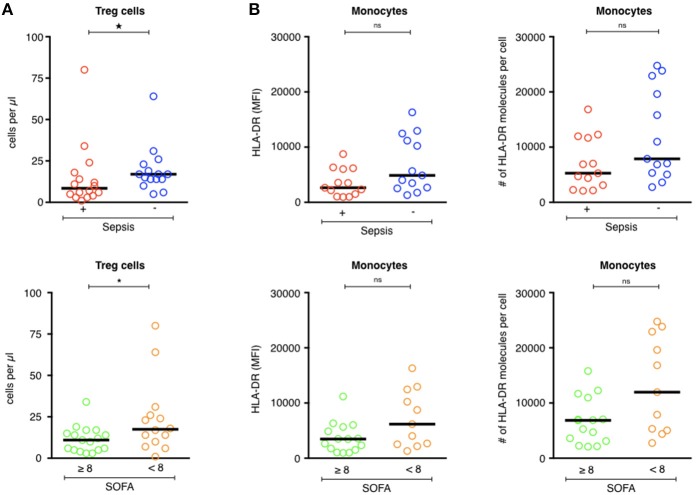
Phenotypic analysis of circulating Tregs and monocytes in ICU patients. (**A**, upper) Comparison of Treg cell counts (cells/μl) of septic patients (red circles) with those of ICU patients without sepsis (blue circles). (**A**, lower) Comparison of Treg cell counts (cells/μl) of patients with severe tissue injuries (green circles) with those of patients with less severe lesions (orange circles). (**B**, upper) Comparison of the MFI of HLA-DR expression and the number of HLA-DR molecules on monocytes of septic patients (red circles) with those of ICU patients without sepsis (blue circles). (**B**, lower) Comparison of the MFI of HLA-DR expression and the number of HLA-DR molecules on monocytes of patients with severe tissue injuries (green circles) with those of patients with less severe lesions (orange circles). The bars show the median. Statistical analyses were performed using the Mann Whitney *U*-test. Differences were considered significant when *P* < 0.05: ^*^P < 0.05. ns: not significant.

Treg lymphocytes are expected to be overrepresented in patients with septic shock and could promote the post-aggression anti-inflammatory response (9, 27). Here, the Treg counts were lower in patients with septic shock than the ICU controls (9/mm^3^ [1–80] vs. 17/mm^3^ [5–64], *p* = 0.04) (Figure 2A, top panel). There was no difference in the number of Treg cells in patients who developed secondary infections and those who did not (11/mm^3^ [3–38] and 14/mm^3^ [1–80], respectively, *p* = 0.67; Table 4). ICU patients who were more severely ill (SOFA ≥ 8) had fewer Treg lymphocytes than those who were less ill (SOFA < 8; 11/mm^3^ [3–34] vs. 17/mm^3^ [1–80], *p* = 0.04; Figure 2A, bottom panel).

#### HLA-DR Expression by Circulating Monocytes

HLA-DR expression on circulating monocytes is frequently used as a marker for the monitoring of immune alterations in critically ill patients, especially those with septic shock (6, 7, 28–31). As expected, HLA-DR expression was lower on circulating monocytes of patients with septic shock (MFI = 2,649 [959–8,752]—HLA-DR Ab/cell = 5,286 [2,089–16,832]) than those of the ICU controls (MFI = 4,882 [1,314–16,298]—HLA-DR Ab/cell = 7,882 [2,760–24,756]; *p* = 0.08; Figure 2B, top panel). There was no difference in HLA-DR expression between patients with secondary infections and those who were uninfected (MFI = 3,702 [959–12,941]—Ab/cell = 6,044 [2,089–24,756] for secondarily infected patients vs. MFI = 3,533 [1,048–16,298]—Ab/cell = 6,959 [2,257–23,846] for patients without secondary infection, *p* = 0.5; Table 4). Less ill patients (SOFA < 8) showed higher HLA-DR expression on their circulating monocytes than those who were more ill (SOFA > 8; Figure 2B, bottom panel), without statistical significance.

#### Circulating ILCs

**Figure 3:**
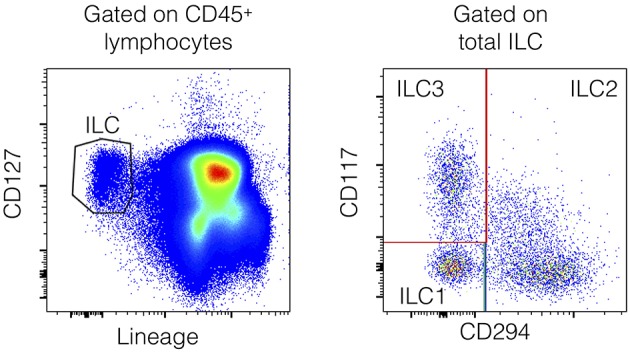
Flow cytometry gating strategy for the identification of human peripheral blood ILCs. ILCs are defined as Lin^−^CD127^+^ cells with a lineage cocktail containing antibodies directed against CD3, CD19, CD14, TCRαβ, TCRγδ, CD94, CD16, FcεRI, CD34, CD123, and CD303 **(Left)**. Within the ILC gate, ILC2s are CD294^+^ cells, whereas ILC1s are CD294^−^CD117^−^ cells and ILC3s are CD294^−^CD117^+^ cells **(Right)**.

Circulating ILCs have never been studied in patients with septic shock. As tissue-resident cells, they represent one of the first immune gates encountered by the infection and participate in the innate immune response through the production and release of pro-inflammatory cytokines (32–39). Total ILCs are defined as lineage-negative CD127^+^ lymphocytes (Figure 3). Among total ILCs, ILC subsets can be discriminated according to the expression of CD294 (CRTH2) and CD117 (cKit): ILC2s are Lin^−^CD127^+^CRTH2^+^ cells, ILC3 are Lin^−^CD127^+^CRTH2^−^CD117^+^ cells, and ILC1 are Lin^−^CD127^+^CRTH2^−^CD117^−^ cells. ILCs in critically ill patients are not spared by the global deficit in circulating lymphocytes. Total ILC counts were slightly lower in both ICU groups than in healthy controls (1,293/mL [483–4,468], 1,242/mL [326–4,085], and 1,632/mL [505–5,846], respectively).

**Figure 4:**
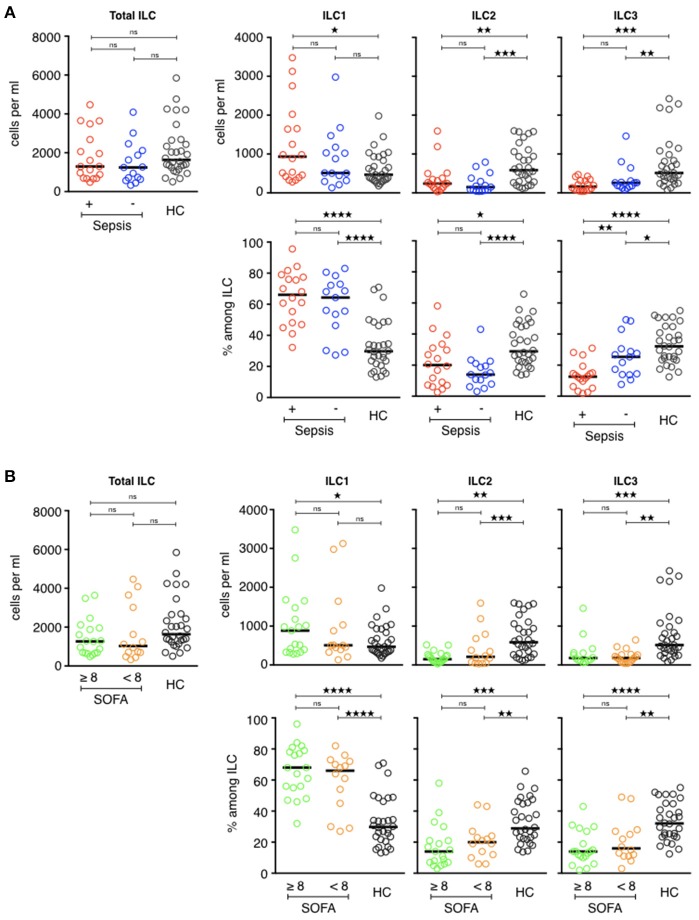
Phenotypic analysis of circulating ILCs in ICU patients. (**A**, upper) Comparison of total ILC, ILC1, ILC2, and ILC3 counts (cells/ml) of septic patients (red circles) with those of healthy controls (dark circles) and ICU patients without sepsis (blue circles). (**A**, lower) Distribution of each ILC subset among total ILCs. (**B**, upper) Comparison of total ILC, ILC1, ILC2, and ILC3 counts (cells/ml) of patients with severe tissue injuries (green circles) with those of healthy controls (dark circles) and patients with less severe lesions (orange circles). (**B**, lower) Distribution of each ILC subset among total ILCs. The bars show the median. Statistical analyses were performed using the Mann Whitney *U*-test. Differences were considered significant when *P* < 0.05: ^*^P < 0.05, ^**^P < 0.01, ^***^P < 0.001, ^****^P < 0.0001. ns: not significant.

However, the ILC distribution was not the same in the two ICU groups (Figure 4A). Septic patients had more circulating ILC1s (931/mL [282–3,478]) than the ICU controls (512/mL [133–2,978]) and, above all, than the healthy volunteers (468/mL [178–1,980], *p* = 0.04). The proportion of ILC1s within total ILCs followed the same pattern: 66% [32–96] of circulating ILC1s in the sepsis group, 64% [27–83] in the ICU controls, and 30% [13-71] in the healthy controls (*p* < 0.0001). Balancing the excess of ILC1s, the ILC2, and ILC3 counts and percentages were significantly lower in both ICU groups than the healthy volunteers. The number of ILC2s in septic patients was 239/mL [29–1,592] (20% [3–58] of total ILC) vs. 147/mL [39–791] (14% [3–43] of total ILCs) in ICU controls and 584/mL [117–1,597] (28% [14–66] of total ILCs) in healthy subjects. The most significant finding for patients with septic shock was a severe deficit in the ILC3 subset (162/mL [41-469] or 13% [2–31]) relative to both the healthy subjects (513/mL [83–2,424], *p* < 0.0001 or 32% [12–55], *p* < 0.0001), and critically ill controls (258/mL [71–1,460], *p* = 0.2 or 25% [8–49] *p* = 0.04). Finally, we compared ILCs and ILC subsets between patients who had a secondary infection and those who did not (Table 4). There was no statistical difference between the two groups based on this criterion. Similarly, there was no difference according to the severity of tissue damage (SOFA ≥ 8 or SOFA < 8) (Figure 4B).

## Discussion

Lymphopenia is common in critically ill patients, especially when the reason for admission is septic shock. The immune deficit following sepsis is called sepsis-induced immunosuppression (25). Other pathological situations, such as trauma (40), or extensive burns (41), can induce the same biological modifications. These situations can all be complicated by “opportunistic” or, more precisely, secondary infections. Here, we confirmed that acute injuries (sepsis, trauma, cardiac arrest) are associated with circulating lymphopenia, which affected all lymphocyte subsets, above all circulating T cells, NK cells, and helper ILCs. Treg lymphocytes were also affected. This appears to contradict previous reports (9), but an examination of the results of Venet et al. show the absolute number of Treg lymphocytes to be lower in septic-ICU patients than in healthy subjects. No ICU controls were considered. Only the percentage of Treg lymphocytes among all CD4^+^ T cells was higher in patients with septic shock, although we did not find this result. The method to identify Treg cells was also not the same as in our study (reagents and samples, whole blood in their study vs. PBMCs in ours). In ICU patients, the deficit in ILCs (as Treg lymphocytes) could be explained by the global lymphopenia.

We also confirmed that lymphopenia is not specific to sepsis. Ischemia-reperfusion (after a cardiac arrest for example), traumatic injuries, or major surgical procedures (42–44) led to the same biological states. Our results are however limited by the small number of patients and our monocentric recruitment. Although “ICU immunosuppression” is not specific to sepsis, it appears to correlate with the severity of the tissue injuries. Comparison of patients with more severe tissue damage (arbitrarily defined by a SOFA score ≥ 8) with those with less severe lesions (arbitrarily defined by a SOFA score < 8) showed that the most ill patients showed a lower count of circulating lymphocytes and lower HLA-DR expression on circulating monocytes. However, there were no major differences in terms of circulating ILCs and their three subsets. It is possible that choosing a SOFA score ≥ 8 to define the severity of tissue injuries may have been somewhat arbitrary. However, the SOFA score adds all organ dysfunction and their intensity. Moreover, the prognosis of these two groups of patients was very different in terms of mortality (2/14 (14%) vs. 9/19 (47%), *p* = 0.046; Table 2). After a severe injury, lymphocyte apoptosis must occur to control inflammation. The initial cytokine storm is subsequent to overstimulation of the innate immune system (45). Pathogen-associated molecular patterns (PAMPs—microbial patterns) and damage-associated molecular patterns (DAMPs—“sterile” patterns) (46) bind to their Toll-like receptors (TLRs) to start an effective immune response to aggression. The resolution of inflammation requires negative feedback involving lymphocyte apoptosis and “tolerization” (47). Lymphocyte apoptosis is the main mechanism leading to lymphopenia in the ICU (40, 48, 49). The more severe the organ damages are, the greater is the deficit in lymphocytes and the resulting so-called “secondary immunosuppression.”

We also observed the absence of a correlation between the depression of biological immune markers (for example, circulating lymphocytes) and the occurrence of secondary infections. This observation contradicts published reports (50, 51). These contradictory findings can be explained by the timing of the blood sample collection. Our biological samples were collected relatively early after the onset of critical illness (24 to 72 h), whereas previous studies used samples taken later (>72 h after the initial infection) (7, 9). It is likely that the risk of secondary infection correlates more with the persistence of an immune deficit than the immune deficit itself, especially in the first hours of care. Even though immunosuppression may be a risk factor (52), the mechanism behind secondary infections appears to be more complex than a simple deficit of several biomarkers (53). In the ICU, comorbidities, length of stay, and the retention of invasive materials can lead to nosocomial infections. Mechanical ventilation can promote VAP and the retention of a central venous catheter can promote catheter-related infections (CRI). For example, in our study, the median length of stay was 18 days [2–60] for secondarily infected patients vs. 4 days [2–120] for uninfected subjects (*p* = 0.001). The length of stay may have been the consequence of secondary infections but an extended length of stay may have also promoted it.

Among all immune modifications, the distribution of circulating ILCs and their subsets appear to show some specificity for the critically ill patients with septic shock. These patients showed a greater proportion of ILC1s balanced by a lower proportion of ILC3s. This result for the ILC1 subset contradicts the recent findings of Cruz-Zárate et al. (49). However, there were differences between their study and ours. Our patients were all in septic shock (infusion of norepinephrine), whereas theirs were mainly in sepsis. Moreover, the distribution of ILCs in their healthy controls is surprising. Although an equal distribution between the ILC subsets has been reported (13, 54), ~85% of the ILCs in their study belonged to the ILC1 subset.

ILC1 is characterized by its ability to produce IFN- γ, which plays a role in the fight against bacterial infections. The ILC1 subset has already been shown to play a role in microbial infections, such as *C. difficile* or *rodentium* (33, 35) colitis and *T gondii* invasion (34) in mouse models. The lack of the ILC3 subset in sepsis suggests that these cells may represent a pool of future and effective ILC1s (ex-ILC3). Lim et al. recently showed (55) that cultured human peripheral blood CD7^+^CD127^+^CD117^+^ cells (ILC3) can give rise to both mature cytotoxic NK and helper ILC subsets, showing an important role for ILC3 in ILC-poiesis (56). ILCs are highly plastic cells (57, 58), which can change phenotype and function depending on their microenvironment. In animals, ILC2s and ILC3s exposed to IL-12 loose the expression of GATA-3 and RORγt, respectively, and acquire features of ILC1s, including T-bet expression and IFN-γ production (59). In the early phase of septic shock, the increase in the pool of ILC1s, originating from ILC3s (ILC1 precursors), may promote the pro-inflammatory response to eliminate the pathogen. Concerning the risk of secondary infections due to the lack of circulating ILCs, our study revealed no significant differences between secondarily infected and uninfected patients, although there was a trend toward a lower proportion of ILC1s and higher proportion of ILC2s in secondarily infected patients (Table 3). ILC2s have been shown to play a role in the resolution of inflammation in ischemic-reperfusion (60) and central nervous system inflammation (61) models. However, they are not “infection models.” In a mouse model of *E. faecalis* infection after burn injury (62), ILC2s had a detrimental role in sepsis and the use of an inhibitor of ILC2 development improved survival. In another study, ILC2s were protective against acute lung injury in a sepsis-induced acute lung inflammation model (63). Shifting the balance in favor of ILC2s (anti-inflammatory) vs. ILC1s (pro-inflammatory) would bear the risk of promoting “immunosuppression” and secondary infections in the ICU.

The study of ILCs in humans is made difficult because of their mostly being located in mucosal tissues (64). Circulating ILCs are considered to be immature relative to tissue-resident ILCs. In humans, the mechanisms by which ILCs circulate between peripheral blood and tissues are still unknown; it is unknown whether a deficit in circulating ILCs is associated with an equal deficiency in tissues. Nevertheless, peripheral blood is the main biological compartment available in humans to analyze the immune response and we show, for the first time, a disequilibrium in the distribution of ILC subsets in patients with septic shock, in which ILCs could participate in the pro-inflammatory immune response and may account for certain immunological post-injury modifications.

## Data Availability

All datasets generated for this study are included in the manuscript/supplementary files.

## Ethics Statement

This study protocol was approved by the Committee for the protection of persons West-North II - France - and the trial was registered online before initiation (NCT03297203).

## Author Contributions

JC and FV devised and supervised the study, designed the research, and wrote the manuscript, with the help of the other co-authors. JC, CP, NB, CF, and CD designed the research, performed experiments, and analyzed the data. JB and MG provided key expertise and samples.

### Conflict of Interest Statement

The authors declare that the research was conducted in the absence of any commercial or financial relationships that could be construed as a potential conflict of interest.
